# Comparison of the effect of a lower versus a higher PEEP strategy on clinically relevant outcomes in invasively ventilated patients without acute respiratory distress syndrome: statistical re-analysis plan of the RELAx trial using a Bayesian framework

**DOI:** 10.62675/2965-2774.20250238

**Published:** 2025-11-10

**Authors:** Alessandro Caroli, Anna Geke Algera, David van Meenen, Marcus J. Schultz, Frederique Paulus, Ary Serpa

**Affiliations:** 1 Austin Hospital Department of Intensive Care Heidelberg Victoria Australia Department of Intensive Care, Austin Hospital - Heidelberg, Victoria, Australia.; 2 Amsterdam UMC Department of Intensive Care Amsterdam Netherlands Department of Intensive Care, Amsterdam UMC, Location AMC - Amsterdam, The Netherlands.

**Keywords:** Critically ill patients, Bayesian analysis, Respiration, artificial, Statistical analysis plan, Positive-pressure respiration

## Abstract

**Background:**

The effect of different levels of positive end-expiratory pressure in invasively ventilated critically ill patients remains a matter of debate. The REstricted *versus* Liberal Positive End-Expiratory Pressure in Patients Without ARDS (RELAx) is a multicentric, randomized trial comparing a lower positive end-expiratory pressure strategy *versus* a higher positive end-expiratory pressure strategy in ventilated patients without acute respiratory distress syndrome, which demonstrated non-inferiority of lower positive end-expiratory pressure compared to higher positive end-expiratory pressure on ventilator-free days. The primary analysis was published in 2020, and a frequentist statistical approach was applied.

**Aim:**

To present the protocol of the Bayesian analysis plan that will be used to re-analyse the RELAx trial to provide complementary and additional insight into this clinical trial.

**Methods:**

This re-analysis will focus on the probability of superiority of the intervention. As an ordinal variable, the primary outcome will be ventilator-free days at day 28, and posterior estimates will be obtained by fitting a hierarchical cumulative logistic regression model. Secondary outcomes will be mortality at day 28, as a binary outcome, and ventilation duration, as a continuous outcome. We will adopt neutral, pessimistic, and optimistic priors informed by current literature, and a fourth prior derived from an expert's survey. Probability thresholds will be defined for superiority, severe harm, and a region of practical equivalence.

**Discussion:**

The RELAx trial findings raise the hypothesis that a lower positive end-expiratory pressure strategy may be at least as effective, if not superior, in specific patient-centred outcomes. This analysis is designed to augment and contextualize the original frequentist analysis of the largest randomized trial comparing positive end-expiratory pressure strategies in non-acute respiratory distress syndrome patients. Results will be presented with a continuum of credible intervals and probabilities of effects to facilitate a nuanced interpretation. We offer clinically meaningful insights that complement and extend the trial's original analysis by reporting probabilities of benefit, harm, and equivalence.

## INTRODUCTION

Mechanical ventilation (MV) is a life-saving intervention for many patients with acute respiratory failure; however, it carries a well-established risk of ventilator-induced lung injury.^(1)^ The combination of low tidal volume (V_t_), low inspiratory plateau pressure (P_plat_), and controlled driving pressure (ΔP) constitutes a cornerstone for lung protective ventilation strategies; conversely, the optimal application of different positive end-expiratory pressures (PEEPs) remains unclear.^(2-4)^

The effects of PEEP on lung aeration result from the prevention of alveolar collapse and recruitment of atelectatic regions, coupled with the risk of overdistension and increased mechanical stress in the already open regions.^(5)^ Furthermore, the extrapulmonary impact of PEEP on cardiac performance may vary according to how the increase in intrathoracic pressure improves lung recruitment, oxygenation, and end-expiratory lung volume.^(6)^ As a result, the delicate balance between the benefit and harm of different values of PEEP could reasonably depend on phenotype and severity of lung illness.

Traditionally, clinical trials have been designed and analysed within a frequentist paradigm, asserting that the evidence for a treatment effect is obtained by testing the null hypothesis of no effect. Under this approach, the p value represents the probability of observing data at least as extreme as those collected, assuming the null hypothesis is true. If the p value falls below the pre-specified threshold of 0.05, the null hypothesis is rejected, and a treatment effect is inferred. On the other hand, a p value above 0.05 is often and imprecisely interpreted as proof of no effect.^(7)^ Such interpretation often results in trials being labelled as "negative" when they should rigorously be defined as "indeterminate", reflecting that the data were insufficient to reject the null hypothesis, rather than confirming an absence of effect.^(8)^

A different paradigm stands behind the Bayesian methodology: to derive the most probable values of the treatment effect (the posterior distribution) enumerating all the possible data sequences that could have happened, selecting those sequences consistent with the observed data (the likelihood) and combining them with specific values derived from the knowledge or beliefs we have in regarding of the treatment effect (the prior distribution).^(9)^ This methodology moves away from the frequentist hypothesis testing. It yields a continuous spectrum of plausible effect sizes along with their probabilities, offering deeper insights than a binary determination of the presence or absence of effect.^(10)^

The REstricted *versus* Liberal Positive End-Expiratory Pressure in Patients Without ARDS (RELAx) is a multicentric, randomized control, noninferiority trial comparing a lower PEEP ventilation strategy *versus* a higher PEEP ventilation strategy on ventilator-free days (VFD) at day 28 in intubated patients without acute respiratory distress syndrome (ARDS).^(11)^ Given the findings of the RELAx trial, which demonstrated non-inferiority of lower PEEP compared to higher PEEP in invasively ventilated intensive care unit (ICU) patients without ARDS, a Bayesian re-analysis is warranted to better characterize the probability of clinical benefit. Although the primary outcome, VFD at day 28, met the non-inferiority criterion, the point estimates favoured lower PEEP, with slightly more VFD in the higher PEEP group (17.7 *versus* 16.7), and lower 28-day mortality (38.4% *versus* 42.0%). These findings raise the hypothesis that lower PEEP may be at least as effective, if not superior, in specific patient-centred outcomes. A Bayesian approach can quantify the probability that lower PEEP improves outcomes, offering a more nuanced interpretation beyond dichotomous non-inferiority conclusions, and may better inform clinical decision-making under uncertainty.

This paper aims to present the protocol for the statistical analysis approach that will be employed to re-analyse the data within the RELAx trial. Bayesian hierarchical modelling will be used to estimate the posterior probability of superiority, inferiority, or equivalence of the lower PEEP against higher PEEP on VFD at day 28 and 28-day mortality. Hypothesis will be assessed to better understand the potential benefit or harm of the intervention, providing a probabilistic and clinically appealing interpretation and allowing for the inclusion of preexisting knowledge into the analysis.

## METHODS

### Study type

Bayesian re-analysis of the RELAx trial. The RELAx trial was registered at www.clinicaltrials.gov (NCT03167580). The study protocol of the original study was approved by the Institutional Review Board of the Academic Medical Center, Amsterdam, The Netherlands, and subsequently published.^(12)^ Patients were included with a deferred informed consent (within 48 hours from randomization), and the principles of the Declaration of Helsinki and the rules of good clinical practice were followed. Due to the nature of the intervention tested, blinding was not possible.

### Design of the original trial

RELAx was an investigator-initiated, national, multicentre, parallel, pragmatic, two-arm randomized control, noninferiority clinical trial conducted in 13 ICUs in the Netherlands between October 2017 and December 2019.^(11)^ It included intubated and ventilated patients with acute respiratory failure expected to be under MV for more than 24 hours without criteria for ARDS diagnosis according to the Berlin definition^(13)^ or severe hypoxemia (partial pressure of oxygen/ fraction of inspired oxygen [PaO_2_/FiO_2_] < 200) and under MV for less than 12 hours. Two ventilation strategies were compared. Patients randomized to the "lower PEEP" group received the lowest PEEP between zero and 5cmH_2_O at which SpO_2_ ≥ 92% or PaO_2_ ≥ 60mmHg, while maintaining FiO_2_ between 0.21 and 0.6; patients assigned to the "higher PEEP" group received a minimum PEEP of 8cmH_2_O to not change it, while FiO_2_ between 0.21 and 0.6, and increase it only in case of hypoxemia and FiO_2_ ≥ 0.6. Safety and rescue manoeuvres in case of severe hypoxemia were implemented for both groups and predefined ventilator weaning strategies. The primary endpoint was the number of VFD at day 28 after ICU admission, and the sample size was powered to demonstrate noninferiority. Secondary endpoints included 90-day mortality, 28-day mortality, ICU-, hospital-mortality, ICU and hospital length of stay, among other clinically relevant outcomes.

### Data management

The RELAx database is protected and de-identified. Data are stored in archives of the Academic Medical Center, Amsterdam, the Netherlands.

### Outcomes

The primary outcome of this analysis will be the primary outcome of the main trial, VFDs at day 28, defined as the number of days that a patient was alive and free of invasive ventilation, calculated from the moment of randomization, if the period of unassisted breathing lasted at least 24 consecutive hours. Patients who died or received invasive ventilation for more than 28 days were considered to have zero VFDs. The secondary outcomes will be the key secondary outcomes of the main trial: 28-day mortality and duration of ventilation among survivors.

### Sample size

All patients included in the RELAx trial will be analysed.

### Analysis plan

Since non-inferiority was already established in the main trial, this Bayesian re-analysis will focus on assessing the probability of superiority. The primary analysis will be based on the intention-to-treat population, in which all the patients will be included and analysed by the treatment group to which they were randomized. The difference in treatment effect between the two groups will be assessed at the individual patient level. Estimates will be obtained by fitting a hierarchical Bayesian cumulative logistic regression model for VFDs as an ordinal outcome, a hierarchical Bayesian logistic regression model for 28-day mortality as a dichotomous outcome, and a hierarchical Bayesian linear regression model for ventilation duration as a continuous outcome.^(14)^ The randomization group will be entered in the model as a fixed effect, and hospitals as random effects. Additional models to evaluate interactions will be fitted, incorporating covariates, interaction terms, and their priors based on clinical relevance. The effect of the intervention on VFDs will be reported as a common odds ratio (OR) with a 95% credible interval (CrI). The cumulative log odds will be modelled such that a parameter greater than zero (or an OR > 1) reflects an increase in the cumulative odds for the VFDs outcome, which implies benefit. The model will assume proportional effects across the ordinal VFDs scale. The effect of the intervention on 28-day mortality will be reported as OR with 95% CrI, and a P (OR < 1) will be assessed as the probability of benefit. The effect of the intervention on ventilation duration will be reported as a mean difference (MD) with 95% Crl, and a P (MD < 0) will be reported as the probability of benefit. All analyses will be performed using R version 4.4.2 (R Core Team, 2016, Vienna, Austria).

### Missing values

Since fewer than 1% of values for the outcome of interest were missing in the original analysis, the primary approach will be a complete-case analysis.

### Prior definition

Previously published recommendations on Bayesian analysis will be followed for defining priors, as well as the methodology from studies applying a Bayesian framework in related clinical fields.^(9,15-17)^

To our knowledge, we identified three meta-analysis relevant to the topic,^(4,18,19)^ each applying similar inclusion criteria, reporting overlapping primary and secondary outcomes, and enrolling comparable populations of non-ARDS ICU patients randomized to low- *versus* high-PEEP ventilation strategies. All three concluded that adjusting PEEP levels had no impact on mortality or duration of MV and did not report VFDs due to its use as an outcome in only one study (Table 1S - Supplementary Material). Based on the current evidence, we assume there is no clear consensus on the optimal PEEP strategy for these patients; if an effect exists, its magnitude is likely small, and neither benefit nor harm can be excluded.

We will adopt a neutral (minimally informative) prior alongside a pessimistic and an optimistic prior to adhere to the literature and span the full range of beliefs on the effect of lower PEEP against higher PEEP, per published recommendations.^(16)^ To enhance the sensitivity of our analysis, we will incorporate an additional prior derived from an online survey eliciting intensive care physicians’ opinions and a priori knowledge on the effect of lower PEEP. A questionnaire containing 27 items was administered to 57 intensivists from the Netherlands. Participants were asked to answer questions from three main domains: doctor and hospital information, punctual estimate, and bonds of usual delivered PEEP and SpO_2_, punctual estimate, and bonds of the 95% confidence interval of the effect of lower PEEP in non-ARDS patients on mortality, VFDs, and duration of ventilation (Item 1S, Table 2S - Supplementary Material).

**Figure 1:**
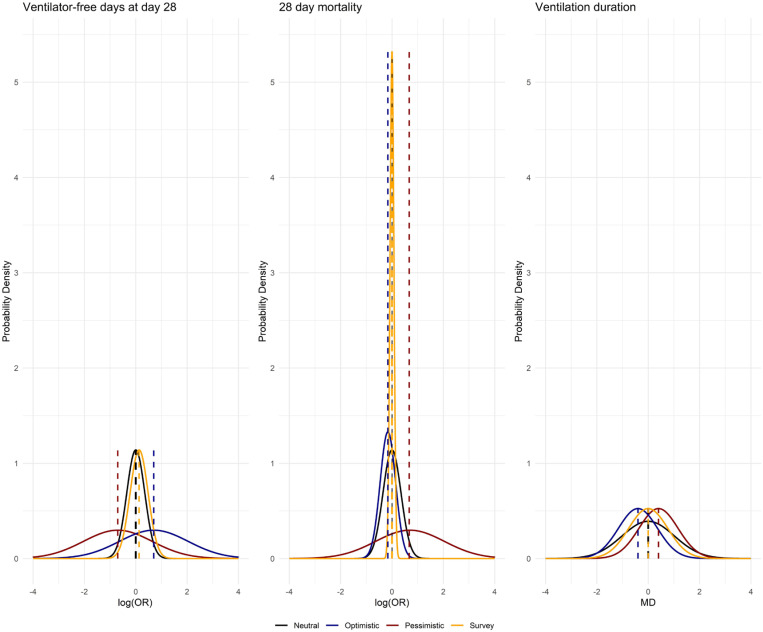
Probability density distribution of priors.

To date, the only randomized trial reporting VFDs in a non-ARDS population is the RELAx trial, with undetermined results regarding the beneficial effects of the intervention. Therefore, we will adopt a reasonable approach that acknowledges the lack of evidence specifically addressing this outcome in the target population and consider the results of the RELAx trial to inform priors distribution. All priors are reported in table 1 and figure 1.

**Table 1:** Priors, region of practical equivalence, superiority, and severe harm thresholds for ventilator-free days and 28-day mortality

Variable	VFDs	28-day mortality	Ventilation duration
Neutral prior	Normal (0, 0.35)	Normal (0, 0.35)	Normal (0, 1.02)
Optimistic prior	Normal (0.7, 1.34)	Normal (-0.16, 0.3)	Normal (-0.4, 0.76)
Pessimistic prior	Normal (-0.7, 1.34)	Normal (0.7, 1.34)	Normal (0.4, 0.76)
Survey prior	Normal (0, 0.6)	Normal (0, 0.075)	Normal (0, 0.76)
ROPE	OR > 0.9 & < 1.1	OR > 0.9 & < 1.1	Not defined
Superiority threshold	OR > 1, posterior probability ≥ 97.5%	OR < 1, posterior probability ≥ 97.5%	MD < 0, posterior probability ≥ 97.5%
Severe harm threshold	OR < 0.75	OR > 1.25	Not defined

VFD - ventilator-free day; ROPE - region of practical equivalence; OR - odds ratio; MD - mean difference.

Probability density distribution of the optimistic (blue), pessimistic (red), neutral (black), and survey (yellow) priors. Dashed lines represent the mean log(odds ratio) and the mean difference.

Priors for the 28-day VFDs outcome will be defined as follows:

1. Neutral (minimally informative) prior is normally distributed and centred at the absence of effect [OR = 1, log(OR) = 0] with a moderate strength such that 95% of prior probability falls in a range of 0.5 ≤ OR ≤ 2. Therefore, our neutral prior will be:
p(θ)=Normal(0,0.35);
2. Optimistic and pessimistic priors will be centred on moderate benefit (OR = 2, log-OR = 0.7) or moderate harm (OR = 0.5, log-OR = −0.7), respectively, with variances selected so that each prior assigns a 30% probability to the opposite outcome. Therefore, our priors will be moderately informative:
$$\begin{array}{c} Optimistic:\;p(\theta )=Normal\;(0.7,1.34)\\ Pessimistic:\;p(\theta )=Normal(-0.7,1.34) \end{array}$$
3. The survey prior is normally distributed. To estimate the distribution parameters, we considered the standardised mean difference (SMD) of the estimated VFDs provided by participants in the lower and higher PEEP groups, as explained in Item 2S (Supplementary Material). This resulted in an elicited SMD of 0.07 between groups with an elicited 95% confidence interval (95%CI) from −0.31 to 0.45. Consequently, we transformed the estimated SMD to log(OR). We built a normal distribution centred at log(OR) = 0.13 with a σ to place 95% of the distribution mass in a log(OR) interval equal to the interval between the lower bound of the elicited 95%CI and the upper bound of the elicited 95%CI:
p(θ)=Normal(0.13,0.35).

Priors for the 28-day mortality outcome will be defined as follows:

1. Neutral (minimally informative) prior is normally distributed and centred at the absence of effect [OR = 1, log(OR) = 0] with a moderate strength such that 95% of prior probability falls in a range of 0.5 ≤ OR ≤ 2. Therefore, our neutral prior will be:
p(θ)=Normal(0,0.35);
2. Pessimistic prior is normally distributed and centred at a moderate harmful effect of lower PEEP [OR = 2, log(OR) = 0.7] with standard deviation (σ) to retain a 30% probability of benefit. The mean effect is informed based on the evidence of one trial reporting a positive effect of higher PEEP in 120 patients with neurological pulmonary edema,^(20)^ and σ is selected to acknowledge the poor quality of the evidence. Therefore, our pessimistic prior will be:
p(θ)=Normal(0.7,1.34);
3. Optimistic prior is normally distributed and centred at weak benefit effect of low PEEP [OR = 0.84, log(OR) = −0.67] with σ to retain a 30% probability of harm. To the best of our knowledge, the only trial reporting a trend in favour of lower PEEP is the RELAx trial in 969 patients [hazard ratio: 0.92 (95%CI: 0.76, 1.11)]. The mean effect is informed based on the odds ratio (OR = 0.84) for 28-day mortality from the RELAx trial, and σ is selected to represent a moderate quality of evidence. Therefore, we will apply the following optimistic prior to inform our analysis:
p(θ)=Normal(−0.16,0.3);
4. The survey prior is usually distributed. To estimate the distribution parameters, we considered the median value of the estimate and bounds provided by participants, as explained in Item 2S (Supplementary Material). This resulted in an elicited mortality in the control group of 29%, an elicited risk difference of 0% between groups, with an elicited 95%CI from −3% to 3%. Consequently, we build a normal distribution centred at the absence of effect [OR = 1, log(OR) = 0] with a σ to place 95% of the distribution mass between a log(OR) equal to the lower bound of the elicited 95%CI and a log(OR) equal to the higher bound of the elicited 95%CI:
p(θ)=Normad(0,0.075).

For the ventilation duration outcome, we will consider a broad and reasonable range of MD in ventilation days to account for the substantial heterogeneity across studies and the prevailing evidence supporting the neutral effect of different PEEP strategies in non-ARDS patients.

Priors for the ventilation duration outcome will be defined as follows:

1. Neutral (minimally informative) prior is normally distributed and centred at the absence of effect (MD = 0) and weak such that 95% of prior probability falls in a range of −2 ≤ MD ≤ 2.
2. Therefore, our neutral prior will be:
p(θ)=Normal(0,1.02);
3. Pessimistic prior is normally distributed and centred at a slight increase in ventilation duration in the low PEEP group, with moderate strength to represent the quality of evidence. The mean effect is informed by the absolute difference reported in the RELAx trial, and σ is selected to retain a 30% probability of benefit. Therefore, our pessimistic prior will be:
p(θ)=Normal(0.4,0.76);
4. An optimistic prior is normally distributed and centred on a slight reduction in ventilation duration associated with lower PEEP. Since no studies published since 2000 have demonstrated a beneficial effect of lower PEEP in non-ARDS patients, the standard deviation was chosen to reflect a moderate level of uncertainty, such that the prior retains a 30% probability of harm. Therefore, our optimistic prior will be:
p(θ)=Normal(−0.4,0.76);
5. The survey prior is usually distributed. We used the median estimate and bounds provided by participants to estimate the distribution parameters to estimate the distribution parameters, resulting in an elicited MD of zero day and an elicited 95%CI from −1 to 2. Accordingly, we will construct a normal distribution centred at the absence of effect (MD = 0) with a σ to ensure that 95% of the distribution mass lies within the elicited 95%CI. Therefore, our survey prior will be (Item 2S - Supplementary Material):
p(θ)=Normal(0,0.76).

**Figure 2:**
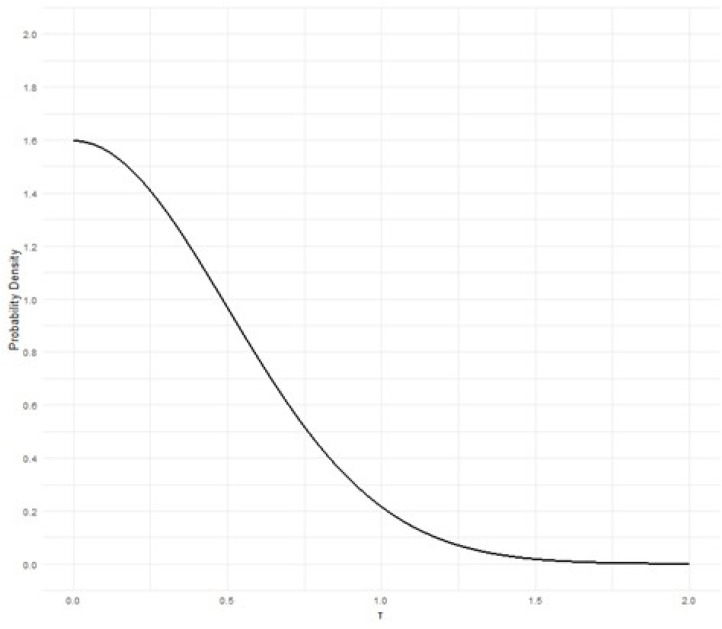
Probability density distribution of the heterogeneity prior.

Being the RELAx trial a multicentric trial conducted within a single country, we will expect a mild degree of heterogeneity between hospitals, thus the random effect prior will be moderately informative and defined as:


$$\tau =\text{Half}-Normal{\left(0,0.5\right)}^{\left(21\right)}\;(\text{Figure 2})$$

Probability density distribution of the heterogeneity prior, τ = Half – *Normal*(0,0.5).

### Credibility interval, range of practical equivalence, and thresholds for superiority and severe harm

Given the absence of validated thresholds for clinical benefit or harm in VFDs and ventilation duration, we acknowledge that defining such thresholds in a Bayesian framework requires careful and transparent justification. Unlike mortality, in which ORs of 1.25 and 0.75 have often been used to denote severe harm or benefit, the interpretation of ORS for ordinal outcomes, such as VFDs, or MD for continuous outcomes, such as ventilation duration, is less well established. For this re-analysis, we will define a threshold for severe harm at OR = 0.75 (log(OR) = −0.29) for VFDs and OR = 1.25 (log(OR) = 0.22) for 28-day mortality. To define a range of practical equivalence (ROPE), we will adopt a range between OR = 0.9 and OR = 1.1 (i.e., −0.1 < log(OR) < 0.1) for both outcomes, representing a range of effect sizes likely to be clinically negligible (Table 1). The ROPE is an arbitrary interval that defines the range of parameter values equivalent to the null value for practical purposes. Consequently, we will apply a decision rule based on how much of the CrI falls outside/inside the ROPE to gather conclusions and their certainty about the null value.^(22)^ These thresholds are, to our knowledge, among the first proposed for VFDs modelled in this way, and we recognize the need for further validation and discussion within the critical care community. Given the lack of clinically meaningful reference points, we will not specify a threshold for severe harm or a ROPE for the MD in ventilation duration.

Finally, we will define the intervention as superior if the posterior probabilities of OR > 1 for VFDs, OR < 1 for 28-day mortality, and MD < 0 for ventilation duration exceed 97.5%. We will also report the posterior probabilities of superiority at the 95% thresholds to enhance interpretability.

### Subgroup analysis

We will perform different subgroup analyses as per the original protocol of the RELAx trial to determine if the relationship between treatment and the primary outcome differs between clinically significant subgroups. This will be done by fitting a Bayesian logistic regression model with an interaction term between treatment and subgroup. We will assess the following subgroups:

1. Diagnosis of cardiac arrest (yes *versus* no);
2. Type of admission (non-surgical *versus* surgical);
3. Reason for intubation (primary respiratory failure *versus* others);
4. Body mass index (> 30 *versus* ≤ 30);
5. PaO_2_/FiO_2_ at enrolment (≤ 200 *versus* > 200);
6. Lung injury prediction score (≥ 4 *versus* < 4); and
7. APACHE score (≥ 86 *versus* < 86).

## DISCUSSION

This paper presents the statistical analysis plan for a Bayesian reanalysis of the data from the RELAx trial. The primary outcome is VFD at day 28, and we will assess the intervention's effect size by fitting Bayesian hierarchical cumulative logistic regression models for the primary analysis and subgroup analysis. Additionally, we will independently evaluate 28-day mortality and ventilation duration to provide a granular understanding of the primary outcome. We will account for between-hospital heterogeneity and include four prespecified prior distributions.

The RELAx trial aimed to test whether using lower PEEP was non-inferior to using higher PEEP in non-ARDS patients. This investigation was conducted in response to epidemiological data showing an increasing tendency to apply higher PEEP levels in mechanically ventilated patients in ICU,^(23)^ and motivated by the rationale that the physiological effects of higher PEEP may be attenuated in patients with less extensive regions of atelectasis and lung collapse, and may differ between those with our without ARDS as well as across the spectrum lung-injury severity.^(3,18)^ In non-ARDS patients, the trial demonstrated that applying lower PEEP is noninferior to applying higher PEEP.

In recent years, Bayesian reanalyses of large randomized controlled trials have become increasingly applied, providing deeper and unanticipated insights, particularly in studies focusing on clinically relevant outcomes that produced indeterminate results under a frequentist approach.^(24-27)^ The Bayesian approach should be considered as complementary to, rather than alternative to, frequentist analysis. By reporting probabilities of intervention effects, it contextualizes outcomes and provides meaningful details to enhance the interpretations of critical care trials.

This analysis has several strengths. First, the RELAx trial is the largest randomized controlled trial in non-ARDS patients. It compares two PEEP strategies, including many patients, and yields high-quality data. We believe that the strength and volume of these data will minimize the impact of prior selection on posterior estimates, reinforcing the robustness of our findings.^(10)^ Second, we present a clear, structured description for selecting priors, following published recommendations and completed by an empirically derived prior from a real-world survey on clinicians’ opinions.^(16,24)^ Third, we will report results in a clinically meaningful manner, providing estimated effects and their probability of benefit, harm, or neutrality, as well as the certainty of our estimates. Fourth, we will conduct sensitivity and subgroup analyses to reinforce our findings.

We acknowledge several limitations of this Bayesian re-analysis. First, Bayesian inference is inherently sensitive to the choice of prior distributions, which can introduce bias. However, we will conduct sensitivity analyses including four distinct priors, including a minimally informative one, and define each prior via a transparent elicitation process to ensure robustness. Second, because this analysis was not pre-planned and will be conducted post-hoc, it inherits all the limitations of the original trial, and the already published results inevitably influence prior beliefs. Lastly, the RELAx trial was conducted in a specific population of non-ARDS patients, limiting the generalizability of its conclusions to a larger cohort.

In conclusion, we aim to present the statistical analysis approach employed to re-analyse the data within the RELAx trial. Using a Bayesian framework as a complementary method will enrich and contextualize the primary frequentist analysis. By interpreting data via a continuum of credible intervals of effects and their probabilities, we will flexibly quantify uncertainty in a clinically relevant manner and provide additional perspectives and insights. Bayesian re-analysis can be a helpful tool to augment the comprehension of critical care trials.

## Supplementary Materials

Supplementary material
