# Supplementary material for: Comparison of the effect of a lower versus a higher PEEP strategy on clinically relevant outcomes in invasively ventilated patients without acute respiratory distress syndrome: statistical re-analysis plan of the RELAx trial using a Bayesian framework
Source: Crit Care Sci. 2025 Nov 10;37:e20250238. doi: 10.62675/2965-2774.20250238 (PMC12977215; doi:10.62675/2965-2774.20250238)
Supplement: Supplementary material [file 2965-2774-ccsci-37-e20250238-suppl01.pdf]

Critical Care Science
https://doi.org/10.62675/2965-2774.20250238

Comparison of the effect of a lower versus a higher PEEP strategy on clinically relevant outcomes in invasively ventilated patients without acute respiratory distress syndrome: statistical re-analysis plan of the RELAx trial using a Bayesian framework

Alessandro Caroli[1], Anna Geke Algera[2], David van Meenen[2], Marcus J. Schultz[2], Frederique Paulus[2], Ary Serpa Neto[1]

Item 1S - Description of the survey conducted to elicit the estimated difference in the effect of low positive end expiratory pressure against high positive end expiratory pressure on commonly used outcomes in ventilation trials in critically ill patients

Between December 1st and December 8th, 2020, a 27-item questionnaire was administered to senior health-care professionals directly involved in the management of critically ill patients.

Purpose of the survey: to obtain opinions about the effect of lowering positive end expiratory pressure (PEEP) on commonly used endpoints in clinical ventilation studies of intensive care patients. Questions refer to intensive care unit (ICU) patients without acute respiratory distress syndrome (ARDS) requiring mechanical ventilation for more than one day.

Instrument: REDCap Survey.

Deanonymization: participants last name was used to compile the participants' list and immediately afterwards removed from the database. The analysis was carried out in a deanonymized dataset without the participants' last name.

QUESTIONS

Part A: Informative questions:

1. What is your last name?
2. What is your current position?
3. How many years have you worked in an intensive care unit?
4. What type of hospital do you work in?

Part B: Questions about the current practice for patients meeting the criteria of the target population:

5. Which PEEP value do you usually use?
6. Which PEEP range do you usually use (lower limit)?
7. Which PEEP range do you usually use (upper limit)?
8. Which oxygenation level do you aim for this patient (SpO_2)?
9. Which oxygenation range do you usually use (lower limit SpO_2)?
10. Which oxygenation range do you usually use (upper limit SpO_2)?
11. At which PEEP do you typically extubate?
12. What do you increase first in the event of desaturation?

This is an open access article under the CC BY license (https://creativecommons.org/licenses/by/4.0/).

Part C: questions about the effect of mechanical ventilation with low PEEP (≤ 5cmH_2O) versus the standard high PEEP (8cmH_2O) in patients meeting the criteria of the target population:

13.a. Estimated VFD28s with low PEEP?

14.b. Estimated VFD28s with high PEEP?

15.Estimated effect size on VFD28s? (a – b)

16.Estimated the precision (95%CI) of the effect size on VFD28s (lower limit)?

17.Estimated the precision (95%CI) of the effect size on VFD28s (upper limit)?

18.c. 28-day mortality with low PEEP (%)?

19.d. 28-day mortality with high PEEP (%)?

20.Estimated effect size on 28-day mortality? (c – d)

21.Estimated the precision (95%CI) of the effect size on 28-day mortality (lower limit)?

22.Estimated the precision (95%CI) of the effect size on 28-day mortality (upper limit)?

23.e. Ventilation duration with low PEEP (days)?

24.f. Ventilation duration with high PEEP (days)?

25.Estimated effect size on ventilation duration? (e – f)

26.Estimated the precision (95%CI) of the effect size on ventilation duration (lower limit)?

27.Estimated the precision (95%CI) of the effect size on ventilation duration (upper limit)?

Item 2S - Description of the method used to compute the parameters of the "Survey Prior" distribution

We assumed that the data from our survey are representative of the current and common opinion in regarding of the delivery of PEEP in mechanically ventilated patients without ARDS and that they are reliable representative of the 'a priori' knowledge to inform a prior distribution. To be consistent with that, our survey prior distribution should be centred on the aggregate result of what the participants expressed as their opinion regarding the risk difference between a ventilation strategy with lower PEEP and a ventilator strategy with higher PEEP in the target population of ICU patients with ARF but not ARDS. Additionally, no previous definition of the strength of the survey should be given, and it should be determined directly from the participants' opinions.

VENTILATOR-FREE DAYS AT DAY 28

To derive the Survey Prior distribution for the odds ratio of ventilator-free days between the intervention and control group and its parameters, we defined:

1. The SMD in ventilator free days between lower PEEP and higher PEEP groups with the Cohen's *d* method:

2. The lower and higher bounds of the 95%CI of the estimated SMD are the computed limits: [-0.45, 0.31].

Under the normality assumption, the standard deviation of the SMD is:

$$SD \approx \frac{upper\ CI - lower\ CI}{2 * 1.96} \approx 0.2.$$

We can transform our SMD to log(OR) and compute μ and σ via the formula:[1]

$$log(OR) \approx \frac{\pi}{\sqrt{3}} * \text{SMD};$$

$$\mu \approx \frac{\pi}{\sqrt{3}} * -0.07 \approx 0.13$$

$$\sigma \approx \frac{\pi}{\sqrt{3}} * 0.19 \approx 0.35;$$

Therefore, our survey prior to 28-VFDs will be:

$$\text{Log}(\text{OR}_{28\text{-VFDs}}) = \text{Normal}(0.13, 0.35).$$

MORTALITY AT DAY 28

To derive the "Survey Prior" distribution for the log(OR) of 28-day mortality between the intervention and control group and its parameters we defined:

1. The control group (high-peep group) mortality risk as the median of the mortality provided by the participant's replies:

2. The mean estimated risk difference in mortality between groups as the median of the provided difference in mortality between the two groups:

3. The lower and higher bounds of the 95%CI of the mean estimated risk difference are the median of the respective limits: [-3, 3].

We derived the intervention group mortality risk interval from the absolute risk difference 95%CI:

$$p1,low = p0 - 0.03 = 0.26,$$
$$p1,high = p0 + 0.03 = 0.32.$$

We converted those risk bounds into OR:

$$\text{ORlow} = \frac{\frac{p1,low}{1 - p1,low}}{\frac{p0}{1 - p0}} = 0.86,$$

$$\text{ORhigh} = \frac{\frac{p1,high}{1 - p1,high}}{\frac{p0}{1 - p0}} = 1.15.$$

We log-transformed:

$$l = log(ORlow),$$
$$h = log(ORhigh).$$

We computed our prior's standard deviation (σ) so that 95% of the mass of the normal distribution (i.e. ±1.96σ) spans between *l* and *h*:

$$\sigma = \frac{h - l}{2\ x\ 1.96} \approx 0.075;$$

Therefore, we will center our survey prior distribution to an OR of 1 (log(OR) = 0) with a SD of 0.075:

$$\log(\text{OR}_{\text{28-day mortality}}) = \text{Normal}(0, 0.075).$$

DURATION OF VENTILATION AMONG SURVIVORS

To derive the "Survey Prior" distribution for the mean difference of ventilation duration between the intervention and control groups and its parameters, we defined:

1. The estimated mean difference as the median of the provided mean difference between groups: MD = 0;
2. The lower and higher bounds of the 95%CI of the mean estimate difference as the median of the respectively provided limits: [-1, 2].

We computed our prior's σ so that the 95% of the mass of the normal distribution (i.e. ± 1.96σ) spans between the lower and upper bounds of the 95%CI:

$$\sigma = \frac{2 - (-1)}{2 * 1.96} \approx 0.76;$$

Therefore, we will center our survey prior distribution to a MD of 0 with a SD of 0.76:

$$\log(\text{MD}_{\text{ventilation duration}}) = \text{Normal}(0, 0.76).$$

Table 1S - Summary of the metanalysis comparing high *versus* low positive end expiratory pressure strategies in intensive care unit patients without acute respiratory distress syndrome

Study	Year	Inclusion criteria	Exclusion criteria	Number of included RCTs	Number of included patients	Low PEEP (range, cmH_2O)	High PEEP (range, cmH_2O)
Pettenuzzo et al.[4]	2021	1. RCTs,<br>2. ICU adult patients<br>3. IMV for reasons other than ARDS,<br>4. Comparing higher PEEP and lower PEEP,<br>5. The primary outcome was hospital mortality	1. Studies not comparing different levels of PEEP with the same ventilator setting	22	2,225	0 - 10	5 - 30
Serpa Neto A et al.[18]	2016	1. RCTs of ventilation,<br>2. ICU adult patients without ARDS at admission,<br>3. Comparing different levels of PEEP,<br>4. Similar tidal volume	1. Observational studies,<br>2. RCTs including patients with ARDS,<br>3. RCTs comparing strategy bundles,<br>4. Other settings than ICU,<br>5. Different PEEP level within one patient	21	1,393	0 - 10	5 - 30
Yi et al.[19]	2022	1. RCTs,<br>2. ICU adult patients without ARDS at admission,<br>3. Randomization to higher against lower level of PEEP<br>4. At least in-hospital mortality and one secondary outcome could be extracted	1. Observational studies,<br>2. Differences in other ventilator settings where enough to affect the outcome,<br>3. Not ICU patients,<br>4. Different PEEP level within a single patient	24	2,307	0 - 10	5 - 30

Study	Outcome (Effect, 95%CI) *			Heterogeneity of the primary outcome	Risk of bias	Key conclusions*
	In-hospital mortality (primary outcome)	28-day mortality	Duration of ventilation			
Pettenuzzo et al.[4]	RR 1.02 [0.89, 1.16], 9 RCTs, 1,502 patients	RR 0.68 [0.33, 1.40], 3 RCTs, 1,152 patients	SMD -0.03 [-0.27, 0.21], RCTs 10, 1,510 patients	$I^2 = 0\%$, $p = 0.62$	Overall high risk, high risk of selection and detection bias	No difference in effect on mortality, improved oxygenation, higher respiratory compliance, lower risk of ARDS occurrence.
Serpa Neto A et al.[18]	RR 0.87 [0.62, 1.21], 7 RCTs, 492 patients, low quality of evidence	RR 0.55 [0.26, 1.18], 3 RCTs, 183 patients, very low quality of evidence	SMD 0.68 [-0.24, 1.61], 3 RCTs, 125 patients, very low quality of evidence	$I^2 = 26\%$, $p = 0.24$	High risk of performance and detection bias	No difference in effect on mortality, improved oxygenation, lower risk of hypoxemia, lower risk of ARDS occurrence.
Yi et al.[19]	RR 0.98 [0.81, 1.19], 9 RCTs, 1,539 patients	RR 0.68 [0.33, 1.40], 3 RCTs, 1,152 patients	SMD -0.30 [-0.64, 0.04], 7 RCTs, 1,393 patients	$I^2 = 14\%$	High risk of performance and detection bias	No difference in effect on mortality, improved oxygenation, lower risk of ARDS occurrence.

RCT - randomized control trial; PEEP - positive end expiratory pressure; ICU - intensive care unit; IMV - invasive mechanical ventilation; ARDS - acute respiratory distress syndrome; 95%CI - 95% of confidence interval; RR - risk ratio; SMD - Standardized Mean Difference.*Effect size and conclusions are reported considering high positive end expiratory pressure as the intervention and low positive end expiratory pressure as reference

Table 2S - Summary of the questionnaire administered during the RELAx survey

	Summary<br>N = 57
ICU specialist	
Anesthesiologist	11 (19)
Cardiologist	1 (1.8)
Internist	21 (37)
Pneumologist	3 (5.3)
Neurologist	1 (1.8)
Fellow	3 (5.3)
Respiratory therapist	8 (14)
Others	9 (16)
Years in ICU	12 (5, 18)
Academic Hospital	28 (49)
Teaching Hospital	24 (42)
Community Hospital	14 (25)
Which usual prescribed PEEP? (cmH_2O)	5 (5, 8)
Which usual lower PEEP limit? (cmH_2O)	5 (5, 5)
Which usual higher PEEP limit? (cmH_2O)	12 (10, 15)
Which usual SpO_2 target? (%)	95 (92, 95)
Which usual lower SpO_2 limit? (%)	90 (90, 92)
Which usual higher SpO_2 limit? (%)	98 (95, 99)
Which usual PEEP at extubation? (cmH_2O)	5 (5, 5)
What increase first in case of desaturation?	
PEEP	10 (18)
FiO_2	47 (82)
Estimated VFD28s in low PEEP group (days)	25 (23, 26)
Estimated VFD28s in high PEEP group (days)	24 (22, 25)
Estimated difference in VFD28s between groups (days)	1 (0, 2)
Estimated 95%CI lower bond of VFD28s difference (days)	-1 (-3, 0)
Estimated 95%CI higher bond of VFD28s difference (days)	3 (1, 5)
Estimated 28-day mortality in low PEEP group (%)	28 (25, 30)
Estimated 28-day mortality in high PEEP group (%)	29 (25, 30)
Estimated difference in 28-day mortality between groups (%)	0 (-2, 0)
Estimated 95%CI lower bond of 28-day mortality difference (%)	-3 (-5, -1)
Estimated 95%CI higher bond of 28-day mortality difference (%)	3 (1, 5)
Estimated ventilation duration in low PEEP group (days)	3 (2, 5)
Estimated ventilation duration in high PEEP group (days)	3 (2, 5)
Estimated ventilation duration difference (days)	0 (-1, 0)
Estimated 95%CI lower bond of ventilation duration difference (days)	-1 (-2, 0)
Estimated 95%CI higher bond of ventilation duration difference (days)	2 (1, 3)

ICU - intensive care unit; PEEP - positive end expiratory pressure; SpO_2 - peripheral oxygen saturation; VDF - ventilator free days; 95%CI - 95% confidence interval. Continuous variables are presented as median ± interquartile range, categorical variables as n (%).

REFERENCE

1. Murad MH, Wang Z, Chu H, Lin L. When continuous outcomes are measured using different scales: guide for meta-analysis and interpretation. BMJ. 2019;364:k4817.
